# Developmental dynamics of cone photoreceptors in the eel

**DOI:** 10.1186/1471-213X-9-71

**Published:** 2009-12-21

**Authors:** Phillippa B Cottrill, Wayne L Davies, Ma'ayan Semo, James K Bowmaker, David M Hunt, Glen Jeffery

**Affiliations:** 1 UCL Institute of Ophthalmology, 11-43 Bath Street, London, EC1V 9EL, UK; 2 Nuffield Laboratory of Ophthalmology, University of Oxford, Level 5-6, West Wing, John Radcliffe Hospital, Headley Way, Oxford, OX3 9DU, UK

## Abstract

**Background:**

Many fish alter their expressed visual pigments during development. The number of retinal opsins expressed and their type is normally related to the environment in which they live. Eels are known to change the expression of their rod opsins as they mature, but might they also change the expression of their cone opsins?

**Results:**

The Rh2 and Sws2 opsin sequences from the European Eel were isolated, sequenced and expressed *in vitro *for an accurate measurement of their λ_max _values. *In situ *hybridisation revealed that glass eels express only rh2 opsin in their cone photoreceptors, while larger yellow eels continue to express rh2 opsin in the majority of their cones, but also have <5% of cones which express sws2 opsin. Silver eels showed the same expression pattern as the larger yellow eels. This observation was confirmed by qPCR (quantitative polymerase chain reaction).

**Conclusions:**

Larger yellow and silver European eels express two different cone opsins, rh2 and sws2. This work demonstrates that only the Rh2 cone opsin is present in younger fish (smaller yellow and glass), the sws2 opsin being expressed additionally only by older fish and only in <5% of cone cells.

## Background

Eels (such as the European eel, *Anguilla anguilla*) change their habitat several times during their life cycle and undergo two distinct metamorphoses that involve morphological, physiological and behavioural changes [1,2]. Eels are catadromous teleosts which are probably spawned in the blue water of the Sargasso Sea [3], possibly at depths of <200 m [3]. The embryo becomes a leptocephalus (translucent leaf-shaped larva) and spends 1-2 years [3] drifting with the Gulf Stream into the North Atlantic. Leptocephali travel the ~6000 km to the European continental shelf with its more green coastal water, where they metamorphose into glass eels, one of two juvenile forms. As elvers (pigmented glass eels) and then as yellow eels (the larger juvenile form), they travel up European rivers and spend 6-20 years in a yellow/brown stained fresh water environment, where they grow and mature as a freshwater species. Subsequently, mature eels must cross the Atlantic Ocean to return to the Sargasso Sea to spawn. Both just prior to and during this migration they undergo 'silvering', usually described as a second metamorphosis but which is more correctly a pubertal event [4], when they become sexually mature adult fish. As sexually mature European eels have never been caught, it is thought that they become fully mature either on the last part of their journey or on reaching their destination. During this life cycle, the photic environment of the eel changes considerably, moving from blue deep sea to green coastal waters, into yellow/brown shallow freshwater and back to blue deep sea again.

Many fish alter their complement of visual pigments during development and this can be attributed in most cases to environmental changes during the life cycle [5-7]. Shallow dwelling fish with access to a wide visual spectrum usually express a full complement of visual opsins, a rod pigment and four cone pigments. Deeper dwelling benthic (living >1 km down) species which have limited or no access to down-welling sunlight have dispensed with many or all of the cone opsins and retained only rod photoreceptors. These often have very extended or multiple layered outer segments for maximal photon catch of attenuated sunlight or bioluminescence [8]. Eels experience more changes to their photic environment than most fish species, and are known to switch the rod visual pigment (*rh1*) on maturation from a "fresh water" form to a "deep-sea" form [9,10]. Wavelength sensitivity is also affected by a change in the ratio of vitamin A1- and A2-derived chromophores used to produce rhodopsin (i.e. vitamin A1-based pigment) and porphyropsin (vitamin A2-based pigment) [11,12]. Maturation is also accompanied by an increase in rod domination in the retina [12,13] arising from the proliferation of rod progenitors [14].

The retinae of most larval teleosts which later undergo a metamorphic event (termed indirect development), contain only cone photoreceptors; with rods being added later after metamorphosis [15-17]. It had been thought that eels were the exception to this rule [13], but work by Omura [18], showed that cones are present in very early (<2 week old) leptocephali. Omura also showed that older leptocephali (>2 weeks) possess a pure rod retina from which the cones must have been lost [18]. However, in postmetamorphic glass eels, the retina contains both rods and cones [19-21]. Eels are therefore known to change the photosensitivity of their rod photoreceptors as well as the photoreceptor composition of the retina during their life cycle. Two spectrally distinct cone classes have been identified [19] but the classes of cone opsin expressed, the timing of cone development, and the distribution of cone types in the retina are largely unknown. This study sets out to determine when changes in cone opsin expression might occur.

## Results

### Cone opsins

Microspectrophotometric (MSP) analysis of the retinae of glass, yellow and silver eels [19] demonstrated the presence of two types of cones, a middle wavelength-sensitive (MWS) class and a short wavelength-sensitive (SWS) class. The former was found at all three developmental stages but showed a variable λ_max _that arose from changes in the proportions of the A1 pigment rhodopsin and the A2 pigment porphyropsin that were present. The λ_max _of the pigment as a pure rhodopsin was estimated to be at 525 nm, consistent with the values found by other workers, allowing for differences in chromophore ratio [20,21]. In contrast, the SWS class was found only in yellow and silver eels. Again, the exact wavelength varied slightly according to the proportions of rhodopsin (A1) and porphyropsin (A2) present, with an estimated λ_max _for pure rhodopsin of 435 nm.

To identify the opsin genes responsible for the pigment present in these MWS and SWS cones, eel retinal cDNA was used. Only two opsin sequences were obtained which corresponded by BLAST analysis to the *rh2 *and *sws2 *opsin coding sequences present in other species. No fragments corresponding to either the *lws *or *sws1 *opsin genes were found.

**Figure 1:**
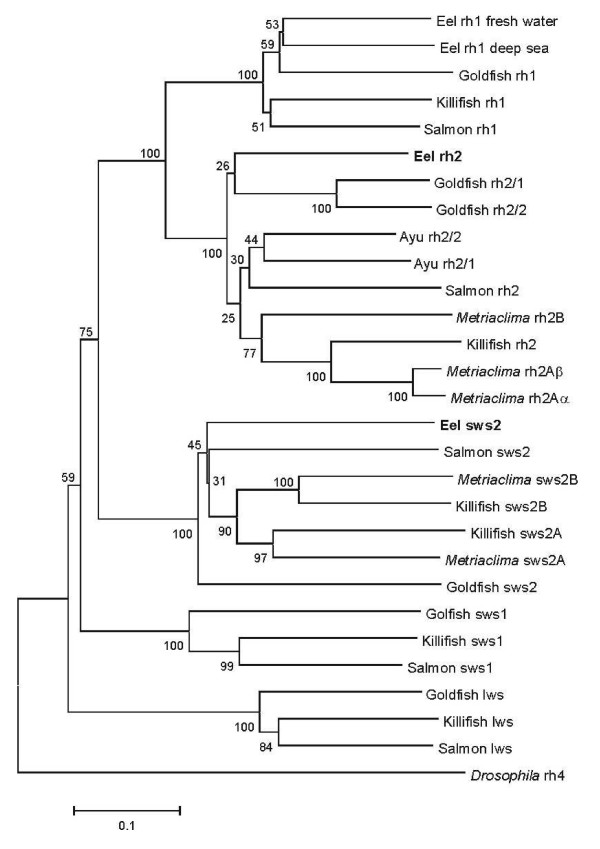
**Phylogenetic tree for visual opsin gene sequences**. The tree was generated by the neighbour-joining method [35], using amino acid sequences aligned by ClustalW [36]. The degree of support for internal branching was assessed by bootstrapping with 1000 replicates using the MEGA2 computer package [37]. The calibration bar is equivalent to 0.1 substitutions per site. GenBank accession numbers for the sequences (from top to bottom) are Eel rh1 "freshwater", L78007; Eel rh1 "deep sea", L78008; Goldfish rh1, L11863; Killifish rh1, AY296738; Salmon rh2, AF201470; Eel rh2, FJ515778; Goldfish rh2/1, L11866, Goldfish rh2/2, L11865; Ayu rh2/2, AB098704; Ayu rh2/1, AB098703; Salmon rh2, AY214132; *Metriaclima zebra *rh2B, DQ0088652; Killifish rh2, AY296739; *Metriaclima zebra *rh2Aβ, DQ088650; *Metriaclima zebra *rh2Aa, DQ088651; Eel sws2, FJ515779; Salmon sws2, AY214134; *Metriaclima zebra *sws2B, AF247118; Killifish sws2B, AY296736; Killifish sws2A, AY296737; *Metriaclima zebra *sws2A, AF247114; Goldfish sws2, L11864; Goldfish sws1, D85863; Killifish sws1, AY296735; Salmon sws1, AY214133; Goldfish lws, L11867; Killifish lws, AY296740; Salmon lws, AY214131; *Drosophila *rh4, NM_057353.

The identity of these sequences was further confirmed using phylogenetic analysis. As shown in Figure 1, the *rh2 *and *sws2 *eel sequences are placed into clades with orthologues from other species. The sequences have been deposited in GenBank with accession numbers of FJ515778 for *rh2 *and FJ515779 for *sws2*.

### Peak sensitivities of cone pigments

**Figure 2:**
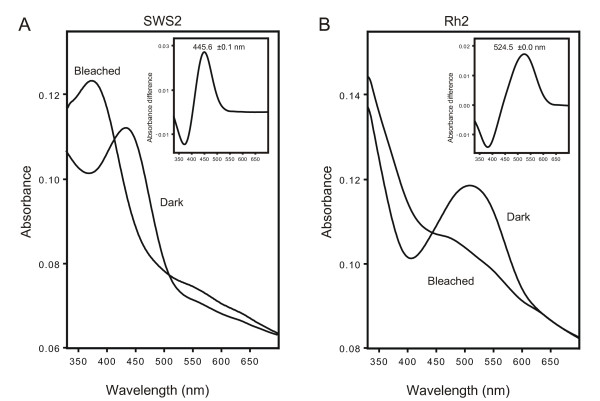
***In vitro *expression of eel cone opsins**. A. Expression of the eel sws2 opsin protein gave a calculated λ_max _of 445.6 ± 0.1 nm, in agreement with the previously measured λ_max _of 435 nm allowing for the differences in chromophores. B. Expression of the eel rh2 opsin protein gave a calculated λ_max _of 524.5 ± 0.0 nm, which is almost identical with the measured λ_max _of 525 nm. The close agreement between the MSP measurements and those of the *in vitro *expressed protein confirms that the isolated opsin sequences are those expressed by the cones.

To correlate the expressed cone opsin genes with the cone classes identified by MSP, full-length eel *rh2 *and *sws2 *sequences were expressed and regenerated with 11-*cis-*retinal, the λ_max _values were obtained by spectrophotometry. As shown in Figure 2, the *rh2 *sequence resulted in a pigment with a λ_max _value of 525 nm, identical therefore to the value obtained by MSP for the MWS cones. The *sws2 *sequence gave a pigment with a λ_max _of 44 6nm, which is somewhat red-shifted compared to the value of 435 nm obtained by MSP for the SWS cones. Such differences may be encountered when comparing between *in vitro *and *in situ *λ_max _values and it was concluded therefore that the MWS and SWS cones express the rh2 and sws2 pigments respectively [5,22].

### Spatial and temporal development of cones classes

**Figure 3:**
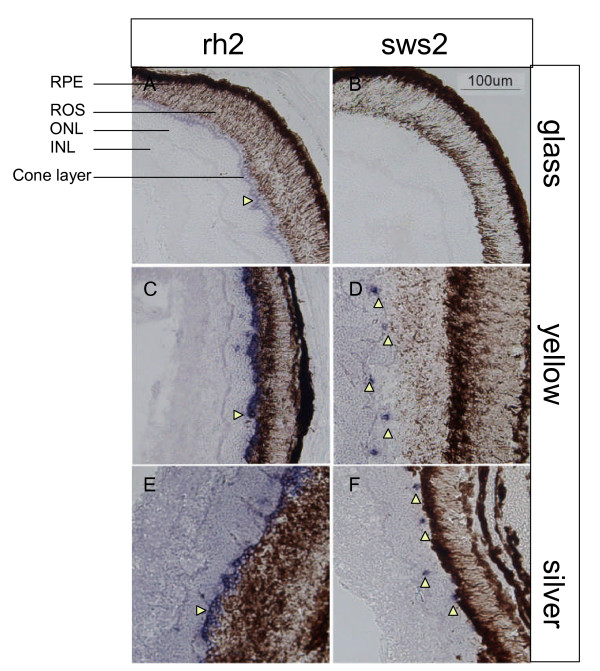
**Distribution of opsin-expressing cone cells in eel retina**. Transverse sections of eel retina from different developmental stages showing localisation of cone opsin expression as detected by *in situ *hybridisation of the 5' end of *rh2 *or *sws2 *opsin mRNA. Cone cell bodies in the eel are arranged in a single layer between the inner nuclear layer (INL) and the rod outer segments (ROS). Glass eel: (A) the layer of cone cells expresses *rh2 *opsin (horizontal arrowhead), (B) *sws2 *opsin expression was not detected in the glass eel retina. Yellow eel: (C) the layer of cone cells expressing *rh2 *opsin (horizontal arrowhead), (D) occasional cone cells expressing *sws2 *opsin (vertical arrowheads). Silver eel: (E) monolayer of cone cells expressing *rh2 *opsin (horizontal arrowhead), (F) occasional cone cells express *sws2 *opsin (vertical arrowheads). Scale bar is 100μm, all panels are to same scale. RPE, retinal pigment epithelium; ROS, rod outer segments; ONL, outer nuclear layer; INL, inner nuclear layer.

*In situ *hybridisation (ISH) was performed with *rh2 *and *sws2 *sense and anti-sense probes. These were derived from the 5' or 3' UTR and coding region of the *RH2 *cDNA, and gave a positive signal in a large proportion of cells in the cone layer. Using the 3' end of the *sws2 *coding sequence a positive signal to the anti-sense probe was observed in a sub-set of cone cell bodies (Figure 3). No signal was obtained for either of the sense (control) probes.

As is usual for fish retinae, the cone cells are arranged as a continuous monolayer situated above the outer limiting membrane, between the outer nuclear layer (ONL, the nuclei of the rod cells) and the rod outer segments themselves (ROS). The signal indicating the presence of *rh2 *opsin mRNA transcript can be seen in all cone cells in the glass eel, and in the majority of cone cells in the yellow and silver eels. The *sws2 *probe also highlighted cones in the monolayer. However, in contrast to the MWS cones, *sws2*-containing cells were restricted to the retinae from older eels, with none in any of the glass eels (20 fish), a few in the elvers/small yellow eels (2 of 19 fish <300 mm in length), but were present in all the larger yellow fish (8 fish >300 mm in length) and in all silver eels (3 animals >770 mm in length). The spatial frequency of the SWS cones is also lower than the MWS cones, with each SWS cone sited at discrete spacings compared to the continuous monolayer of MWS cones. The distance between SWS cones varied widely (partly according to fish size), from a value of 1 in 5 found in several mid-sized yellow eels, to 1 in 12 cells in one of the silver eels. Most mid-sized yellow eels measured showed average MWS cone spacings of 7 ± 1.7 μm; whilst for the SWS cones, the same fish gave values of 45 ± 15 μm. The silver and very large yellow eels were more variable (50-90 μm between SWS cells, with standard deviations of up to 40%, typically 68 ± 23 μm). For mid-sized yellow eels, this was a linear spacing of approximately one in every 6-7 cones, equivalent to an array frequency of approximately 1 in 30.

### Relative levels of opsin expression

#### Cone pigments

**Figure 4:**
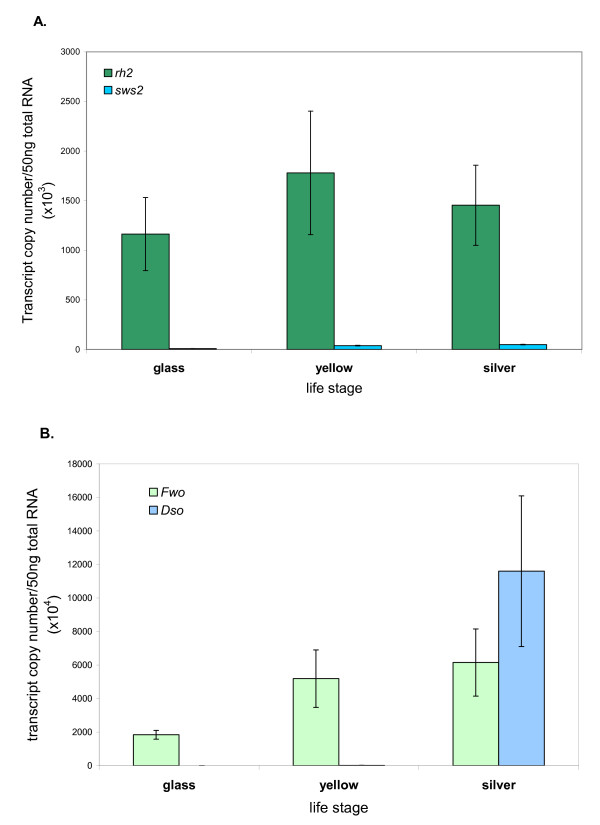
**Changes in expression levels of opsins at different life stages of the eel**. A. The expression level of *rh2 *and *sws2 *cone opsin transcripts as copy numbers per 50 ng total RNA. The level of green opsin is approximately equivalent in the different life stages, but the amount of blue opsin doubles between the glass and silver stage. B. The expression of the two rod (*rh1*) opsin transcripts as copy numbers per 50 ng total RNA. The level of fwo transcript appears to increase slightly between the glass and later stages, but the amount of dso transcript increases markedly between the glass and silver life stages. Error bars show standard deviations which are necessarily large for the non-glass eels as each sample represents a point on a continuum.

Quantification of the levels of *rh2 *and *sws2 *opsin transcripts by qPCR are in general agreement with the findings of the *in situ *hybridisation experiments, as is shown in Figure 4A. The level of *rh2 *opsin mRNA was slightly lower in glass eels than in yellow and silver eels, where the levels were very similar. A surprising finding was the presence of expressed *sws2 *opsin mRNA in glass eels when no SWS2-containing cones were evident by *in situ *hybridization staining. Across all three stages, the level of *sws2 *message was substantially lower than *rh2 *message, consistent with the relative paucity of SWS2-containing cones compared to MWS cones.

#### Rod pigments

It has been previously shown [12] that the sexual maturation of both the European and American eel is accompanied by an increase in rod domination of the retina and a shift in absorbance maxima of the rod photoreceptors to a shorter wavelength; this shift is the result of a switch in expression in rod opsin from the "freshwater" (fwo) to the "deep sea" (dso) form [9,10]. The presence of this switch was confirmed by qPCR (Figure 4B) where expression of the dso form, although just detectable in glass and yellow eels, is significant only in silver eels where it represents around 60% of the total expressed rod opsin. The levels of the fwo form show a significant increase from glass to yellow stage, and this level is maintained in the silver eels despite the simultaneous expression at a higher level of the dso form.

**Figure 5:**
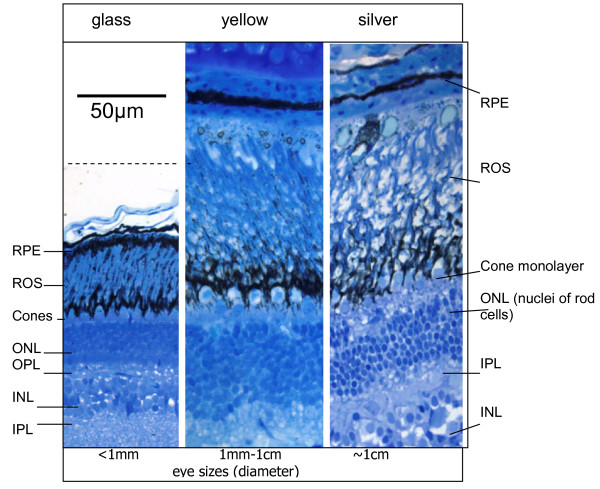
**Changes in the retina in different life stages of the eel**. 2 μm thick plastic-embedded sections of eel retina from different life stages were stained with Richardson's Stain. It can be clearly seen that the thickness of the retina, and especially of the outer nuclear layer (rod cell nuclei), doubles between the glass and the silver stages of the life cycle. The size of the eye itself changes dramatically between the different stages, as indicated in the bottom panel, increasing ten-fold in diameter. Such changes must necessitate large scale remodelling of the retinal components. RPE, retinal pigment epithelium; ROS, rod outer segments; ONL, outer nuclear layer; INL, inner nuclear layer; OPL, outer plexiform layer; IPL, inner plexiform layer. Scale bar = 50 μm, all histology panels are to the same scale.

The ratio of cone to rod opsin expression reflects the rod dominant nature of the eel retina. Even at the glass eel stage, a ratio of rod to cone (*rh2 *+ *sws2*) opsin mRNA expression is 17:1, and this rises to 30:1 in yellow eels and to 121:1 in silver eels (with expression of fwo and dso forms of rod opsin combined). Morphologically (Figure 5), rods contribute an increased proportion of the retina in yellow eels compared to glass eels, with an approximate doubling in the thickness of the outer nuclear layer. This increase does not however continue into the silver eel stage, so the substantial increase in the rod to cone opsin ratio seen at this stage is likely to reflect an increase in the relative production of rod opsin mRNA during the switch over from fwo to dso forms.

## Discussion

We have demonstrated here that the European eel (*Anguilla anguilla*) changes opsin gene expression more times during its lifetime than had previously been reported. It was shown by Omura [18] that cones cells are present in very early leptocephali; whilst older leptocephali possess a pure rod retina [23]. It was also shown by Hope *et al*. [9], and quantified by Zhang *et al*. [10], that rod photoreceptors start to express the dso form of rod opsin as the animal undergoes puberty (silvering) before migration from fresh to salt water. Glass eels have a mixed retina with rh2 opsin expressed in cones [19], indicating that they have already made a change from the pure rod retina of the leptocephali. This probably occurs as they move from the deep sea into the shallower waters of the continental shelf and estuaries. A second change in cone opsin expression then occurs as they either move into the rivers or remain longer in the estuaries. Instead of a monolayer of cone photoreceptor cells expressing only the rh2 pigment, occasional sws2 opsin-expressing cones appear within the monolayer. It could not be determined whether an rh2 opsin-expressing cone changes to express sws2 opsin (as happens during the switch in expression from fwo to dso forms of rod opsin [9,10]); or if new cones which express the sws2 pigment are inserted at intervals into the monolayer. The result appears as an array of SWS-expressing cones interspersed within the MWS cone monolayer.

The results of the qPCR experiments did not fully confirm the findings of the *in situ *hybridisations on the varying amounts of sws2 cone opsin in the different life stages of the eel. Whilst no cells containing *sws2 *opsin mRNA were detected by *in situ *hybridisation in the glass stage eels, *sws2 *opsin sequence was detected in these samples by qPCR, which is commonly believed to be a more sensitive detection method. From the *in situ *hybridisation results, *sws2 *opsin expression did not appear to start until well into the yellow stage (greater than 300 mm length), and continued at a similar level into the silver stage. However, qPCR, suggested that the levels of *sws2 *opsin expression in the yellow and silver eel was only about 4 to 5-fold higher than in the glass eel stage, as can be seen in figure 4A. A possible explanation for this could be that *sws2 *opsin message is transcribed as a default at a very low level by all cone cells, but at too low a level to be detected by *in situ *hybridisation. It is thought that expression of the *sws1 *opsin gene is the default pathway for mouse cone cells [24] and it could be that the *sws2 *message is the default in eel cones. In this case, after the eel reaches a certain size, the *sws2 *opsin transcript is turned off in most cells which continue to express *rh2 *opsin and are spectrally identifiable as MWS cones, but is upregulated in specific cells which then become detectable by *in situ *hybridisation as *sws2*-expressing cells and by microspectrophotometry as SWS cones. The calculated transcription copy numbers suggested that the level of *rh2 *expression was approximately 40-fold higher for all stages than that for *sws2*, which is in good agreement with the apparent 30-fold fewer *sws2 *opsin-expressing cells seen by *in situ *hybridisation.

There are two possible mechanisms for the changes in opsin gene expression. The most probable mechanism is that the existing cone cell changes the opsin type it is expressing. This is already known to occur in the rod cells of eels when they switch from expression of fwo to expression of dso [25]. It has also been shown to occur in rainbow trout [26], where single cones change from expression of *sws1 *to *sws2 *opsin. Alternatively, newly generated photoreceptors expressing the sws2 opsin could be generated from Müller glial-associated retinal progenitors that can function as multipotent retinal stem cells [26] and inserted into the photoreceptor layer [27].

## Conclusions

In this paper, we have shown that European eels express only two classes of cone opsin, rh2 and sws2. The rh2 class is expressed in young animals, whereas the sws2 opsin is expressed only by older animals. Spectral analysis of *in vitro *expressed pigments demonstrates that their absorbance peaks correspond to the values obtained by *in situ *MSP for the MWS and SWS cones. This was further confirmed by *in situ *hybridization where it was shown that a class of rh2-expressing cones is present as a monolayer in the retina at the glass eel stage. The sws2-expressing cones appear later at the yellow eel stage as occasional cells within this monolayer. The presence of two cone classes at the later stages of pre-pubertal development is consistent with a relatively broad-spectrum light environment present in the rivers and estuaries that the eels occupy prior to full sexual maturation and migration into the deep ocean.

## Methods

### Animals

European eels (*Anguilla anguilla L*.) were obtained from different sources according to their size. Glass eels were obtained from http://www.glasseel.com. Elvers and small yellow eels were collected by the authors from the river Thames, with the permission of the Environment Agency. Larger yellow eels were obtained from a registered Thames eel fisherman (Gary Hillar). Very large yellow and silver eels were obtained from Billingsgate fish market. All animals were treated in line with the guidelines laid out in the code of practice for the housing and care of animals used in scientific procedures (Animals (Scientific Procedures) Act 1986). Animals that were not used immediately on arrival were maintained in large aerated tanks with natural light or, if in a dark room, on a 12L:12D light cycle using fluorescent aquarium light tubes. Immediately before use animals were anaesthetised by immersion in MS222 (1:2000 w/v in water) before being culled following standard procedures. Mature silver status was assessed by eye and body length measurements which were fed into the formula of Pankhurst [28], and only those animals with a Eye Index scores >6.5 were considered true silver eels.

### Identification of gene sequences

Total RNA was isolated from the heads of glass eels, or eyecups (lacking lenses) of slightly larger animals or the retina/RPE of large animals, using Tri Reagent (Sigma). The mRNA was transcribed into cDNA using the SMART RACE Amplification kit (Clontech Laboratories Inc) according to the manufacturer's instructions. Opsin sequences were PCR-amplified using degenerate primers designed to conserved areas of opsin sequences. The 5' and 3' ends were obtained by RACE using the anchor primers in the SMART RACE cDNA Amplification kit paired with specific primers designed to previously isolated sequence.

### *In situ *hybridisation

Eyes isolated from animals that had been culled following standard procedures, were fixed overnight in 4% paraformaldehyde, transferred to 30% sucrose and the lens removed (from larger eyes). After overnight incubation in sucrose they were snap frozen in OCT compound (Vector Labs) and stored at -80°C before being sectioned on a cryostat.

Probes were prepared from 3' and 5' opsin sequences (5' or 3' UTR and coding region of the *rh2 *cDNA; and the 3' end of the *sws2 *coding sequence) cloned into the pGEM T_(easy) _vector (Promega). The inserts were made into riboprobes labelled with DIG using the SP6/T7 Transcription kit (Roche Ltd.). The DIG-labelled probes were hybridised to 10μm frozen tissue sections and hybridised probe was visualised using BM purple (Roche Ltd.). Sections were examined using an Olympus BX50 microscope and photographs taken with a Nikon digital camera DXM 1200.

### qPCR

The RNA extraction protocol followed was as above using Tri Reagent (Sigma) or Trizol (Invitrogen), with mRNA transcribed into cDNA using Superscript III (Invitrogen) with oligo d(T) primer (Invitrogen). First strand cDNA was prepared from 1 μg total RNA extracted from heads of glass eels, or eyecups lacking lenses of larger fish. The visual pigments were quantified using gene-specific primers; which were designed, using the primer 3 program on the NCBI website, to amplify 300 bp fragments. Internal controls (housekeeping genes) used were as previously published: mitochondrial cytochrome b (intron-free) and acidic ribosomal phosphoprotein P0 (ARP) from Weltzein *et al*. [29], and glucose 6 phosphate dehydrogenase (g6pdh) and 6-phosphogluconate dehydrogenase (6pgdh) from Pierron *et al*. [30]. A minimum of three reactions were performed on a minimum of three animals of each of the three sizes (24 glass, 18 yellow and 4 silver). Raw data was analysed using the DART-PCR spreadsheet [31], and R_0 _values calculated. For normalisation purposes a normalisation factor was calculated from the R_0 _values of internal control genes using geNORM [32], and the resulting values used to normalise the results obtained for the test genes. Standard curves were plotted using cloned genes and their calculated copy numbers for the reactions in parallel with the test samples.

### *In vitro *opsin expression

This was performed as previously described [33,34]. The full-length coding region of the *rh2 *and *sws2 *opsins were isolated by PCR using specific primers and cloned into the eukaryotic expression plasmid pMT4. The resulting plasmid was used to transiently transfect HEK-293T cells. The recombinant visual pigments were extracted and column purified with the Rho1D4 antibody. Pigments were regenerated by incubation with 11-*cis*-retinal, and analysed by a Spectronic Unicam UV500 dual-beam spectrophotometer. After 3 independent recordings, pigments were bleached by exposure to bright fluorescent light for 30 minutes and re-analysed. The bleached pigment spectra were subtracted from the dark spectra to produce a difference spectrum and a peak absorbance (λ_max_) value using standard computer programs. The resulting visual spectra were overlaid by visual pigment templates and best-fit spectral curves were obtained.

### Histology

Eyes for embedding in plastic were fixed in fresh 2% formaldehyde, 2% glutaraldehyde in PBS, dehydrated through an ascending alcohol series and embedded in Technovit 7100 resin (Heraeus), as per manufacturer's instructions. After the blocks had hardened, sections were cut at 2 μm on a microtome using a glass knife before being stained with Richardson's stain and mounted in DPX.

## Authors' contributions

All authors have read and approved the final manuscript. The *in vitro *expression work was done by WLD who generated figure 2 and provided degenerate primers for the *lws *and *sws1 *sequences. MS obtained the sequence of the *rh2 *opsin, PBC isolated the *sws2 *sequence, performed the ISH and qPCR, and initially drafted the manuscript. DMH generated figure 1 and modified the manuscript; GJ, DMH and JKB obtained the funding, and guided the project and write-up of this manuscript.
